# Strain-Based Monitoring Methodology and Numerical Validation for the Evaluation of Transverse Connection Condition in Precast Multi-Girder Bridges

**DOI:** 10.3390/s26134043

**Published:** 2026-06-25

**Authors:** Wenhao Zheng, Han Wei, Jiehua Jiang, Wanheng Li

**Affiliations:** Research Institute of Highway, Ministry of Transport, Beijing 100088, China; wh.zheng@rioh.cn (W.Z.); jh.jiang@rioh.cn (J.J.); wh.li@rioh.cn (W.L.)

**Keywords:** structural health monitoring, precast multi-girder bridge, damage detection, response correlation, signal separation

## Abstract

Precast multi-girder bridges are widely utilized in highway infrastructure but are susceptible to transverse connection deterioration, which can lead to single-girder load-bearing failures. Existing structural health monitoring methods based on the correlation of total dynamic strain responses often fail to identify early-stage damage due to the static masking effect, where dominant, in-phase quasi-static components overshadow subtle, damage-sensitive dynamic features. To overcome this limitation, this paper proposes a novel condition indicator based on the correlation of high-frequency dynamic strain increments. An online streaming processing pipeline is developed, incorporating automated single-vehicle crossing event extraction, frequency-targeted signal decoupling, and indicator smoothing. Theoretical derivations on a dual-beam model demonstrate that the proposed indicator is a structural-intrinsic metric, exhibiting high sensitivity to joint stiffness while remaining robust against variations in vehicle weight and speed. Numerical simulations on an 8-slab finite element bridge model under stochastic traffic flow further verify the effectiveness of the framework. Results indicate that the proposed indicator can localize both progressive degradation and sudden brittle failures. Additionally, the method maintains reliability down to a signal-to-noise ratio of 30dB and robustness to hyper-parameter selection. While the current framework is established based purely on numerical validation and has not yet been tested using real bridge strain data, it shows numerical feasibility and provides a solid theoretical and algorithmic foundation for the automated condition evaluation of precast multi-girder bridges, supporting future field validation for both long-term maintenance and emergency response.

## 1. Introduction

**Figure 1:**
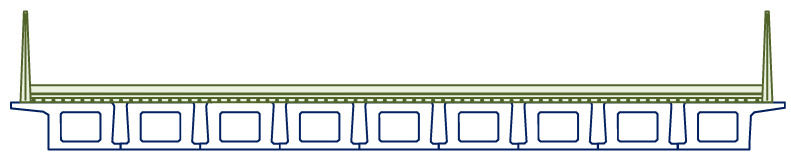
Typical cross-section of precast multi-girder bridges.

Precast multi-girder bridges (PMGBs), as schematically illustrated in Figure 1, have been universally adopted in short-to-medium-span highway infrastructures globally due to their cost-effectiveness, standardized prefabrication, and rapid on-site assembly [1,2]. In these bridge systems, the transverse connections—typically constructed as longitudinal concrete hinge or wet joints—serve as the critical load-transfer mechanism to ensure that multiple precast girders function collaboratively. However, these joints have proven to be the most vulnerable components of the structure under the long-term coupled effects of massive cyclic traffic loads, material shrinkage, and environmental erosion. The degradation of joints fundamentally alters the transverse load distribution, eventually precipitating the dangerous “single-girder load-bearing” phenomenon. In this critical state, an individual overloaded precast girder behaves in isolation, leading to concrete crushing, reinforcement yielding, and potentially catastrophic bridge collapses [3,4]. Therefore, ensuring the structural integrity is of paramount importance for the safety and reliability of PMGBs.

To this end, extensive research has been conducted on the offline condition assessment of transverse connections. For instance, Xiao et al. [5] performed field tests and presented a modified hinge-connected slab method to estimate the actual stiffness of hollow slab beams and hinge joints. Similarly, a hybrid framework combining physical models and vision-based measurements was proposed in [6] for joint damage detection and condition assessment. Moreover, advanced non-contact techniques utilizing computer vision and optical flow methods have been recently developed to extract dynamic responses and accurately identify structural modal parameters [7]. Other approaches include damage identification utilizing finite element (FE) analysis combined with load testing results [8], and techniques that extract mode shapes from the dynamic responses excited by test vehicles [9].

While these offline, periodic methods provide intuitive visual or localized structural information, they are fundamentally plagued by significant drawbacks. Manual inspections are labor-intensive, highly subjective, and heavily dependent on the inspector’s empirical expertise. Furthermore, static load testing necessitates severe traffic disruptions and yields only a discrete, snapshot-like evaluation of the structural condition. Most critically, these intermittent offline methods are intrinsically incapable of capturing sudden structural deterioration in real time, thereby failing to provide timely early warnings before catastrophic events occur.

To overcome the limitations of periodic inspections, real-time structural health monitoring (SHM) systems leveraging continuously streaming sensor data have emerged as a paradigm shift. Wen et al. [10] proposed an index based on the Pearson correlation coefficients (PCCs) of strain measurements to characterize the transverse collaborative working performance of PMGBs. Dan et al. [11] further demonstrated the accuracy and applicability of this method using real-bridge monitoring data. However, this index, extracted directly from the total dynamic strain, has proven to be insensitive to early-stage damage due to the static masking effect. Alternatively, methods based on transverse strain or displacement mode shapes have been explored for transverse connection damage detection [12,13]. Nevertheless, these techniques largely remain within the realm of short-term or small-batch data analysis, highlighting the pressing need for continuous, long-term data utilization strategies. Recently, comprehensive studies on transverse connection condition monitoring using displacement spectrum similarity have been conducted across theoretical, numerical, and real-bridge investigations [14,15]. A notable drawback of this displacement spectra indicator is its narrow sensitivity margin to joint stiffness variations; the indicator merely drops from 1.0 to 0.84 as the joint degrades from a perfectly intact state to complete failure. Shang et al. [16] proposed a stiffness-estimation methodology for the condition evaluation of hinge joints. While this approach provides an intuitive physical metric, its implementation relies on the precise prior of structural parameters (e.g., mass, elastic modulus, mode amplitude), which inevitably introduces parameter uncertainties and compromises the evaluation accuracy.

To dismantle these limitations, this paper proposes an online, long-term dynamic-increment indicator framework for PMGBs that eliminates the static masking effect, delivers a widened sensitivity margin, and operates purely on operational strain data without necessitating any structural parameter priors. The core contribution lies in decoupling the damage-sensitive dynamic increments from the energy-dominant quasi-static baselines, thereby mitigating the static masking effect. By integrating well-established classical techniques—including automated event extraction, Ormsby filtering, PCC, and moving average smoothing—the proposed method extracts a structural-intrinsic dynamic-increment indicator that is sensitive to joint stiffness while remaining robust against traffic variations. The entire algorithm is structured as an online, point-by-point streaming pipeline. Although the proposed method has not yet been validated using real bridge strain data and relies purely on numerical simulations at this stage, this framework not only provides robust, external-insensitive quantitative metrics for long-term progressive damage monitoring but also establishes a numerical basis for future emergency warning systems against sudden severe failures.

The remainder of this paper is organized as follows: Section 2 establishes the analytical dual-beam governing equations and theoretically proves the existence of the static masking effect. Section 3 details the mathematical formulation of the proposed dynamic-increment indicator alongside the real-time streaming signal processing framework. Section 4 provides a proof-of-concept validation based on the analytical model, demonstrating the indicator’s intrinsic robustness to stochastic traffic parameters. Section 5 conducts FE simulations under varying scenarios to verify the damage identification capability of the proposed framework and its numerical feasibility for future emergency warning applications. The robustness against measurement noise and hyper-parameter selection is further validated. Finally, the main findings are summarized in Section 6.

## 2. Analytical Formulation of the Dual-Beam System

A simplified analytical dual-beam system is established in this section to deliver a fundamental, mechanism-oriented proof of concept, which serves as a minimum-viable conceptual baseline. Its purpose is to mathematically expose the static masking effect that plagues the traditional total-strain indicator and to theoretically prove the damage-sensitive nature of the proposed dynamic-increment indicator. The multi-beam system that mimics the real-world bridge will be accommodated and validated through FE numerical experiments in Section 5.

### 2.1. Governing Equations of the Dual-Beam System

**Figure 2:**
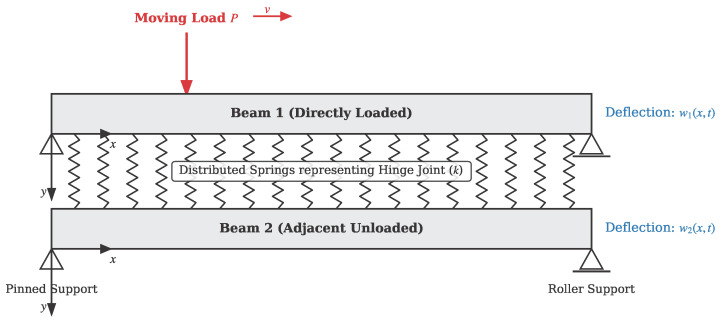
Kinematic schematic of the continuous dual-beam model subjected to a moving vehicle load.

Two precast girders connected by a hinge joint can be modeled as a dual-beam system. As illustrated in Figure 2, two parallel simply supported Euler–Bernoulli beams (denoted as Beam 1 and Beam 2) with an identical span length *L*, unit mass *m*, and flexural rigidity EI are considered. The hinge joint between the two beams is simplified as continuously distributed linear springs with a stiffness of *k*. A moving vehicular load, idealized as a constant point load *P*, travels along Beam 1 at a constant velocity *v*. Damping is neglected in this theoretical derivation to obtain a clear analytical solution.

Based on D’Alembert’s principle, the governing partial differential equations for the dynamic deflections $w_{1}\left(x,t\right)$ and $w_{2}\left(x,t\right)$ of the two beams can be expressed as

(1) $$EI\frac{\partial^{4}w_{1}\left(x,t\right)}{\partial x^{4}}+m\frac{\partial^{2}w_{1}\left(x,t\right)}{\partial t^{2}}+k\left[w_{1}\left(x,t\right)-w_{2}\left(x,t\right)\right]=P\delta \left(x-vt\right)$$

and

(2) $$EI\frac{\partial^{4}w_{2}\left(x,t\right)}{\partial x^{4}}+m\frac{\partial^{2}w_{2}\left(x,t\right)}{\partial t^{2}}+k\left[w_{2}\left(x,t\right)-w_{1}\left(x,t\right)\right]=0,$$

where δ(·) is the Dirac delta function, *x* is the longitudinal coordinate, and *t* is the time.

### 2.2. Modal Decoupling and Analytical Solution

The coupled equations can be decoupled by introducing the symmetric and antisymmetric deflections, which can be formulated as

(3) $$w_{s}\left(x,t\right)=w_{1}\left(x,t\right)+w_{2}\left(x,t\right)$$

and

(4) $$w_{a}\left(x,t\right)=w_{1}\left(x,t\right)-w_{2}\left(x,t\right),$$

respectively. Adding and subtracting Equations (1) and (2) yields two independent equations:

(5) $$EI\frac{\partial^{4}w_{s}}{\partial x^{4}}+m\frac{\partial^{2}w_{s}}{\partial t^{2}}=P\delta \left(x-vt\right),$$

(6) $$EI\frac{\partial^{4}w_{a}}{\partial x^{4}}+m\frac{\partial^{2}w_{a}}{\partial t^{2}}+2kw_{a}=P\delta \left(x-vt\right).$$

Using the Galerkin method, the structural responses are expanded into modal series:

(7) $$w\left(x,t\right)=\sum_{n=1}^{\infty }q_{n}\left(t\right)\sin\left(\frac{n\pi x}{L}\right).$$

Substituting Equation (7) into Equations (5) and (6) and integrating over the beam length [0,L] yields the ordinary differential equations for the *n*-th modal generalized coordinates $q_{sn}\left(t\right)$ and $q_{an}\left(t\right)$,

(8) $${\ddot{q}}_{sn}\left(t\right)+\omega_{sn}^{2}q_{sn}\left(t\right)=\frac{2P}{mL}\sin\left(\Omega_{n}t\right),$$

(9) $${\ddot{q}}_{an}\left(t\right)+\omega_{an}^{2}q_{an}\left(t\right)=\frac{2P}{mL}\sin\left(\Omega_{n}t\right),$$

where $\Omega_{n}=\frac{n\pi v}{L}$ is the driving frequency of the moving load. The natural frequencies of the symmetric and antisymmetric modes are denoted as $\omega_{sn}$ and $\omega_{an}$, respectively, which are given by:

(10) $$\omega_{sn}={\left(\frac{n\pi }{L}\right)}^{2}\sqrt{\frac{EI}{m}},$$

(11) $$\omega_{an}=\sqrt{\omega_{sn}^{2}+\frac{2k}{m}}.$$

Assuming zero initial conditions (i.e., q(0)=0 and $\dot{q}\left(0\right)=0$), the analytical solutions for the generalized coordinates are derived as:

(12) $$q_{sn}\left(t\right)=\frac{2P}{mL(\omega_{sn}^{2}-\Omega_{n}^{2})}\left[\sin\left(\Omega_{n}t\right)-\frac{\Omega_{n}}{\omega_{sn}}\sin\left(\omega_{sn}t\right)\right],$$

(13) $$q_{an}\left(t\right)=\frac{2P}{mL(\omega_{an}^{2}-\Omega_{n}^{2})}\left[\sin\left(\Omega_{n}t\right)-\frac{\Omega_{n}}{\omega_{an}}\sin\left(\omega_{an}t\right)\right].$$

In Equations (12) and (13), the structural response is decomposed into two distinct physical parts. The first term within the brackets represents the *quasi-static component*, which is dictated by the moving frequency of the vehicle load ($\Omega_{n}$) and reflects the macro-deformation that follows the load’s position. The second term represents the *dynamic increment*, which is governed by the structural dynamic characteristics (i.e., the natural frequencies $\omega_{sn}$ and $\omega_{an}$) and represents the inertial oscillation excited by the moving force.

The dynamic deflections can be reconstructed by superimposing the symmetric and antisymmetric generalized coordinates:

(14) $$w_{1,2}\left(x,t\right)=\frac{1}{2}\sum_{n=1}^{\infty }\left[q_{sn}\left(t\right)\pm q_{an}\left(t\right)\right]\sin\left(\frac{n\pi x}{L}\right).$$

### 2.3. Dynamic Strain Responses

According to the Euler–Bernoulli beam theory, the relationship between the longitudinal strain ε(x,t) at a vertical distance *y* from the neutral axis and the beam deflection w(x,t) is governed by the structural curvature:

(15) $$\varepsilon \left(x,t\right)=-y\frac{\partial^{2}w\left(x,t\right)}{\partial x^{2}}.$$

By substituting Equation (14) into Equation (15), the dynamic strain at the bottom of Beam 1 and Beam 2 is formulated as:

(16) $$\varepsilon_{1,2}\left(x,t\right)=\frac{y}{2}\sum_{n=1}^{\infty }{\left(\frac{n\pi }{L}\right)}^{2}\left[q_{sn}\left(t\right)\pm q_{an}\left(t\right)\right]\sin\left(\frac{n\pi x}{L}\right).$$

It is noteworthy that the symmetric generalized coordinate $q_{sn}\left(t\right)$ is entirely independent of the hinge joint stiffness *k*. From a mechanical perspective, during a purely symmetric deformation, the adjacent beams experience identical deflections, resulting in zero relative displacement between them. Consequently, the hinge joint remains mechanically inactive and transmits no internal shear forces.

In contrast, the stiffness *k* is exclusively embedded within the antisymmetric natural frequency $\omega_{an}$, which in turn governs the antisymmetric generalized coordinate $q_{an}\left(t\right)$. For conciseness, define the amplitude coefficients for the symmetric and antisymmetric modes as:

(17) $$A_{sn}=\frac{2P}{mL(\omega_{sn}^{2}-\Omega_{n}^{2})},\;A_{an}=\frac{2P}{mL(\omega_{an}^{2}-\Omega_{n}^{2})}.$$

These coefficients dictate the behaviors of the transverse load distribution under two extreme scenarios:

- Ideal Rigid Connection (k→∞): When the hinge joint acts as a perfectly rigid body, the antisymmetric frequency $\omega_{an}$ approaches infinity. Consequently, both $A_{an}$ and $q_{an}\left(t\right)$ converge to zero. The structural response is completely dominated by the symmetric component $q_{sn}\left(t\right)$, indicating that the dual-beam system degenerates into a single equivalent beam moving in absolute unison.
- Complete Hinge Damage (k→0): When the transverse connection completely fails, the loss of lateral constraint causes the antisymmetric frequency to degenerate into the symmetric frequency ($\omega_{an}\to \omega_{sn}$). Under this condition, $q_{an}\left(t\right)\to q_{sn}\left(t\right)$. According to Equations (14) and (16), the dynamic response of Beam 2 identically approaches zero, physically representing a complete loss of transverse load transfer capability.

By substituting Equations (12) and (13) into Equation (16), and evaluating at the mid-span (x=L/2), the total dynamic strain can be reorganized into a quasi-static component $\varepsilon^{qs}\left(t\right)$ and a dynamic increment component $\varepsilon^{dyn}\left(t\right)$:

(18) $$\varepsilon_{1,2}\left(t\right)=\varepsilon_{1,2}^{qs}\left(t\right)+\varepsilon_{1,2}^{dyn}\left(t\right),$$

where the quasi-static strain components are given by:

(19) $$\varepsilon_{1,2}^{qs}\left(t\right)=\frac{y}{2}\sum_{n=1,3,\ldots }^{\infty }{\left(-1\right)}^{\frac{n-1}{2}}{\left(\frac{n\pi }{L}\right)}^{2}\left[A_{sn}\pm A_{an}\right]\sin\left(\Omega_{n}t\right),$$

and the dynamic strain increments are formulated as:

(20) $$\varepsilon_{1,2}^{dyn}\left(t\right)=-\frac{y}{2}\sum_{n=1,3,\ldots }^{\infty }{\left(-1\right)}^{\frac{n-1}{2}}{\left(\frac{n\pi }{L}\right)}^{2}\left[A_{sn}\frac{\Omega_{n}}{\omega_{sn}}\sin\left(\omega_{sn}t\right)\pm A_{an}\frac{\Omega_{n}}{\omega_{an}}\sin\left(\omega_{an}t\right)\right].$$

**Figure 3:**
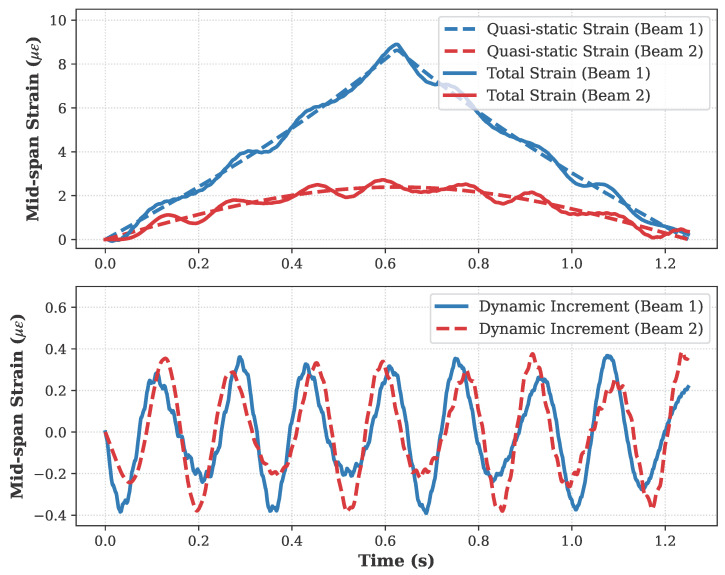
Typical time history of mid-span strain responses revealing the masking effect and asynchronous dynamic increments.

A time-history simulation of the mid-span strain responses for a dual-T-girder system subjected to a moving load, using the parameters detailed in Table 1, is shown in Figure 3. The result illustrates three fundamental physical mechanisms:

- Low-pass filtering effect of the hinge joint. As illustrated in the upper subplot, the quasi-static component of Beam 1 exhibits a sharp, triangle-like trend. This characteristic stems from the contribution of high-order modes. Conversely, the quasi-static response of Beam 2 manifests as a smooth, sine-like wave, which is governed by the structural filtering mechanism. According to the frequency relationship $\omega_{an}=\sqrt{\omega_{sn}^{2}+2k/m}$, the natural frequency squared of high-order modes dominates the transverse stiffness term ($\omega_{sn}^{2}\gg 2k/m$), resulting in $\omega_{sn}\approx \omega_{an}$. Consequently, the amplitude coefficient for Beam 2, $\left[A_{sn}-A_{an}\right]$, decays rapidly toward zero for higher-order components. This ensures that the hinge joint acts as a low-pass filter, blocking the transmission of high-order quasi-static components from the loaded beam and resulting in a simplified, sine-like profile in the adjacent beam.
- Dominance of the quasi-static component. The amplitude of the quasi-static strain reaches approximately 10 με, whereas the dynamic strain increment fluctuates within a mere ±0.4 με. This orders-of-magnitude difference empirically proves that the quasi-static component dominates the total strain energy.
- Amplitude–phase asynchrony in dynamic increments. Unlike the quasi-static baselines that synchronize in overall trend, the dynamic increments of the two beams exhibit an amplitude–phase asynchrony. This decorrelation is caused by the opposite signs (±) of the antisymmetric components, which forces the wave amplitude and phase of Beam 2 to shift in opposite temporal directions. As hinge joint stiffness degrades, the increasing antisymmetric modal energy exacerbates this asynchrony.

## 3. Proposed Indicator and Signal Processing Framework

### 3.1. Limitation of the Previous Indicator

In the previous study [10], the hinge joint condition is evaluated using the correlation of the total dynamic strain responses between adjacent precast beams. Given the dynamic strain signals of mid-span points $\varepsilon_{i}\left(t\right)$ and $\varepsilon_{j}\left(t\right)$ from adjacent beams *i* and *j*, the PCC is employed to represent the correlation:

(21) $$\rho_{ij}=\frac{\mathrm{cov}(\varepsilon_{i},\varepsilon_{j})}{\sqrt{D\left(\varepsilon_{i}\right)\;\cdot \;D\left(\varepsilon_{j}\right)}},$$

where cov(·) denotes the covariance, and D(·) represents the variance of the signal. Considering the inherent stochastic nature of actual traffic loads (e.g., varying vehicle weights, speeds, and arrival times) in operational environments, $\rho_{ij}$ obtained within a time window exhibits significant randomness. To eliminate this load-induced uncertainty and extract a structural-intrinsic parameter, the previous study proposed utilizing the average of the correlation coefficients over *N* observation windows as the hinge joint condition indicator:

(22) $${\bar{\rho }}_{ij}=\frac{1}{N}\sum_{k=1}^{N}\rho_{ij}^{\left(k\right)}.$$

As derived in Section 2.3, when the transverse connection stiffness *k* decreases, the increasing energy of the antisymmetric mode exacerbates the amplitude–phase asynchrony between $\varepsilon_{i}^{dyn}\left(t\right)$ and $\varepsilon_{j}^{dyn}\left(t\right)$, which inherently leads to a reduction in their covariance $\mathrm{cov}(\varepsilon_{i}^{dyn},\varepsilon_{j}^{dyn})$. Consequently, the PCC $\rho_{ij}$ calculated from the total dynamic strain responses decreases as the structural integrity of the hinge joint degrades.

However, the practical sensitivity of this indicator is severely compromised by the *static masking effect* of the quasi-static component. Given the significant frequency gap between the quasi-static and dynamic increment components, their covariance can be neglected ($\mathrm{cov}(\varepsilon^{qs},\varepsilon^{dyn})\approx 0$). Thus, the total covariance of the strain signals can be expanded as $\mathrm{cov}\left(\varepsilon_{i},\varepsilon_{j}\right)\approx \mathrm{cov}\left(\varepsilon_{i}^{qs},\varepsilon_{j}^{qs}\right)+\mathrm{cov}\left(\varepsilon_{i}^{dyn},\varepsilon_{j}^{dyn}\right)$. Similarly, the variance of each signal is approximated as $D\left(\varepsilon \right)\approx D\left(\varepsilon^{qs}\right)+D\left(\varepsilon^{dyn}\right)$. Substituting these relationships into Equation (21) yields:

(23) $$\rho_{ij}\approx \frac{\mathrm{cov}\left(\varepsilon_{i}^{qs},\varepsilon_{j}^{qs}\right)+\mathrm{cov}\left(\varepsilon_{i}^{dyn},\varepsilon_{j}^{dyn}\right)}{\sqrt{\left[D\left(\varepsilon_{i}^{qs}\right)+D\left(\varepsilon_{i}^{dyn}\right)\right]\left[D\left(\varepsilon_{j}^{qs}\right)+D\left(\varepsilon_{j}^{dyn}\right)\right]}}.$$

As analyzed in Section 2.3, the structural response is largely governed by the quasi-static component induced by vehicle weights, i.e., $D\left(\varepsilon^{qs}\right)\gg D\left(\varepsilon^{dyn}\right)$. Furthermore, the quasi-static components of adjacent beams synchronize in their overall trend, resulting in a large covariance ($\mathrm{cov}(\varepsilon_{i}^{qs},\varepsilon_{j}^{qs})$).

According to Equation (23), this high-energy, correlated quasi-static baseline acts as a dominant masking term in both the numerator and the denominator. Consequently, the subtle, damage-induced drop within the dynamic covariance is heavily diluted. This mathematical structure suppresses the indicator’s sensitivity, rendering the previous indicator based on the total dynamic strain inherently insensitive to hinge joint degradation.

### 3.2. Proposed Indicator Based on Dynamic Increments

To overcome the inherent limitation imposed by the masking effect, the dominant quasi-static baseline must be stripped away from the total dynamic response. By isolating the dynamic strain increments $\varepsilon_{i}^{dyn}\left(t\right)$ and $\varepsilon_{j}^{dyn}\left(t\right)$ from adjacent beams *i* and *j*, the dynamic-increment PCC is defined as:

(24) $$\rho_{ij}^{dyn}=\frac{\mathrm{cov}(\varepsilon_{i}^{dyn},\varepsilon_{j}^{dyn})}{\sqrt{D\left(\varepsilon_{i}^{dyn}\right)\;\cdot \;D\left(\varepsilon_{j}^{dyn}\right)}}.$$

Consistent with the statistical framework of the previous method, to eliminate the uncertainty stemming from random traffic loads and keep the indicator structure-intrinsic, the final proposed indicator is defined as the average of the dynamic-increment PCCs obtained over *N* observation windows:

(25) $${\bar{\rho }}_{ij}^{dyn,(k)}=\frac{1}{N}\sum_{k=1}^{N}\rho_{ij}^{dyn,(k)}.$$

Unlike the previous indicator ${\bar{\rho }}_{ij}$ governed by Equation (23), the proposed indicator ${\bar{\rho }}_{ij}^{dyn}$ is effectively isolated from the dominant quasi-static components, $\mathrm{cov}(\varepsilon_{i}^{qs},\varepsilon_{j}^{qs})$ and $D(\varepsilon^{qs})$. Consequently, the reduction in the dynamic covariance $\mathrm{cov}(\varepsilon_{i}^{dyn},\varepsilon_{j}^{dyn})$, which is caused by the amplitude–phase asynchrony and the surging antisymmetric modal energy during hinge joint degradation, will be explicitly reflected in the calculated coefficient. This theoretically makes the new indicator a sensitive metric for evaluating the transverse connection condition.

### 3.3. Extraction of the Proposed Indicator from Operational Data

For practical engineering implementation, strain sensors can be deployed on the bottom at the mid-span cross-section of each individual slab. For a bridge comprising *M* parallel slabs, a total of *M* sensors are aligned along the same transverse mid-span centerline. Within the streaming pipeline, any two adjacent sensors (Sensor *i* and Sensor i+1) are automatically grouped to form a pairing loop. By continuously evaluating the dynamic-increment indicator of each adjacent pair, the condition of each joint can be independently isolated and tracked.

In real-world SHM systems, the raw strain signal $\varepsilon^{raw}\left(t\right)$ is a complex superposition of multiple physical processes, including environmental effects, quasi-static responses, dynamic increments, and measurement noise. To implement the proposed indicator ${\bar{\rho }}_{ij}^{dyn}$ under operational traffic, the following signal processing framework is developed.

#### 3.3.1. Automated Single-Vehicle Crossing Event Extraction

Processing a continuous data stream is computationally inefficient and introduces environmental noise into the indicator calculation. To autonomously isolate the core vehicle-on-bridge duration $T_{c}=\left[t_{start},t_{end}\right]$, a straightforward state-transition triggering method is implemented. The identification procedure consists of three sequential steps:

1. Continuous MWA calculation: The moving window average (MWA) is continuously computed for the raw strain signal of every instrumented precast girder to quantify the quasi-static envelope.
2. Global threshold triggering: To account for the transverse load distribution, the maximum MWA across all girders at each time step is extracted to form a global energy envelope. A pattern-matching logic is then applied to this envelope using a predefined threshold:
  - If the previous MWA is below the threshold and the current MWA exceeds it, the start time of the current window is designated as the vehicle entry time ($t_{start}$).
  - Conversely, if the previous MWA exceeds the threshold but the current MWA falls below it, the end time of the previous window is designated as the response conclusion time ($t_{end}$).
3. Single-peak detection: The time interval $\left[t_{start},t_{end}\right]$ determined by the above criteria is extracted as a candidate single-vehicle crossing event. A peak detection algorithm is then applied to the MWA within this window. To isolate the genuine vehicle-induced wave, three constraints are enforced on the local maxima: (1) a height threshold (30% of the maximum envelope); (2) a minimum temporal distance (0.1 s); and (3) a prominence threshold (10% of the maximum). If a single distinct peak is identified, the segment is classified as a single-vehicle crossing event and retained for subsequent processing. If multiple peaks are detected (indicating overlapping vehicle platoons), the entire segment is discarded. This strict exclusion is necessary because overlapping vehicles introduce complex, multi-source transient excitations that corrupt the phase relationships of the structural dynamic response, which would distort the subsequent frequency-targeted filtering process.

#### 3.3.2. Frequency-Targeted Extraction of Dynamic Increments

To isolate the damage-sensitive dynamic increments $\varepsilon^{dyn}\left(t\right)$ from the extracted crossing events, a frequency-targeted Ormsby filter [17] is employed. The Ormsby filter is defined by specifying the passband and stopband frequencies, which inherently forms a transition band Δf (configured to 0.2 Hz in this study). Unlike digital filters (e.g., standard rectangular windowed filters) that suffer from severe Gibbs ringing, this smooth transition band significantly minimizes edge ripples and prevents phase distortion in the time domain. This is a critical requirement since the proposed correlation-based indicator relies fundamentally on the phase synchrony between adjacent girders.

To eliminate boundary effects (edge transients) introduced by finite-length signal filtering, an extended physical windowing strategy is adopted. Specifically, an additional 10% temporal buffer of actual recorded operational data is appended to both ends of the core crossing duration $T_{c}=\left[t_{start},t_{end}\right]$.

The Ormsby filtering is sequentially applied to this extended data array. This band-pass filtering process simultaneously eliminates both the low-frequency quasi-static components induced by vehicle weight and the ultra-low-frequency temperature-induced strain variations. To rigidly remove these unwanted low-frequency elements while minimizing the attenuation of the bridge’s first-order vibration mode, the lower cut-off frequency of the band-pass filter can be set between 0.8 and 0.9 times the bridge’s fundamental frequency. Concurrently, since the dynamic vibration energy of simply supported girder bridges is predominantly governed by the lower-order modes, the upper cut-off frequency can be set slightly higher than the third-order vertical bending frequency. This configuration ensures that the primary dynamic structural responses are preserved while effectively rejecting irrelevant high-frequency measurement noise. Finally, by truncating the 10% temporal buffers, the purely structural dynamic increments are reconstructed with high fidelity, ensuring that the responses at the boundaries are formulated based on genuine structural states without numerical artifacts.

#### 3.3.3. Online Algorithmic Framework for Indicator Calculation

The calculation of the proposed indicator ${\bar{\rho }}_{ij}^{dyn}$ is implemented as a continuous, point-by-point streaming architecture, which operates as follows:

1. **Continuous data collection & preprocessing:** Raw strain signals are continuously acquired from the instrumented precast girders and instantly processed using the data anomaly detection and cleaning framework [18,19,20]. Importantly, baseline shifts and spikes must be cleaned for the subsequent MWA calculation.
2. **Vehicle crossing monitoring:** As each clean strain data point $\varepsilon^{raw}\left(t\right)$ arrives, the MWA is updated. An automated triggering logic continuously monitors this MWA against a predefined threshold to identify the entry and exit times of vehicles.
3. **Extended event extraction:** Once a crossing event concludes (indicated by the MWA dropping below the threshold for a brief margin to ensure complete vehicle departure), the system instantly extracts the core event array. Additional pre-event and post-event data segments are seamlessly appended to both ends of the core duration to form an extended extraction window.
4. **Single-vehicle validation:** The extracted event is then evaluated using the peak-counting algorithm. If overlapping multi-peak responses are detected, the event is discarded to prevent phase distortion; otherwise, it is validated as a strict single-vehicle crossing event.
5. **Signal separation:** The validated signal is sequentially passed through the targeted Ormsby filters to strip away the quasi-static baseline and high-frequency noise. The previously appended pre- and post-event data segments are then truncated to yield the dynamic increments $\varepsilon^{dyn}\left(t\right)$ without filtering edge artifacts.
6. **Online correlation:** The PCC of the dynamic increments is calculated for each pair of adjacent girders to obtain the instantaneous coefficient $\rho_{ij}^{dyn,(k)}$ for the *k*-th valid event.
7. **Indicator update:** The moving ensemble average of the PCCs over the most recent *N* valid events is updated instantaneously, yielding the stable indicator ${\bar{\rho }}_{ij}^{dyn,(k)}$. The system then waits for the next structural excitation.

## 4. Theoretical Model Validation

In this section, the analytical model formulated in Section 2 is employed for a proof-of-concept study. The primary objective is to demonstrate that the dynamic-increment PCC between adjacent beams is intrinsic to the shear-key stiffness and remains insensitive to the vehicle load in an idealized state.

### 4.1. Analytical Dual-Beam Model

The bridge is modeled as two parallel Euler–Bernoulli beams. The function of the joint can be modeled via a continuous layer of elastic springs [10,14,16,21]. A concentrated load is assumed to traverse the first beam at a constant velocity. The default parameters for the precast T-girder and the vehicle can be found in Table 1.

### 4.2. Sensitivity Analysis to Joint Stiffness

**Figure 4:**
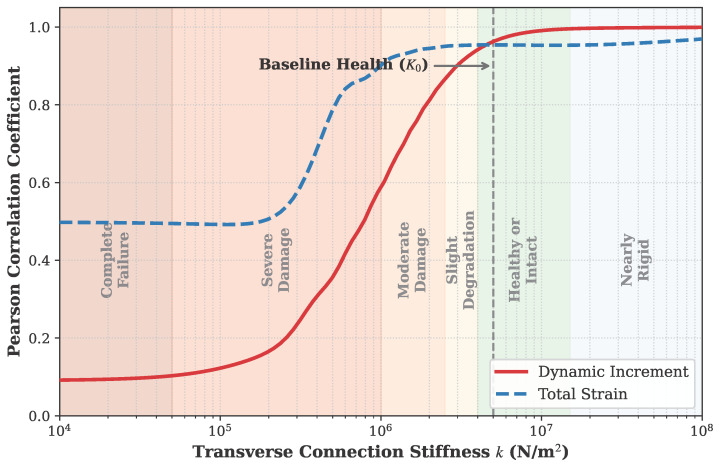
Evolution of the correlation with respect to hinge stiffness across different health states. The red solid line denotes the proposed dynamic increment correlation, while the blue dashed line represents the traditional total strain correlation. The shaded backgrounds indicate the corresponding structural health states.

In theoretical and numerical analysis, joint damage is generally modeled by the reduction in transverse connection stiffness [6,10,13,14,21]. In these studies, the joint stiffness in an intact state is empirically smaller than 10^7^ N/m^2^. Consequently, 10^8^ N/m^2^ represents a nearly rigid-body connection (more than 10 times the intact state); conversely, the lower bound of 10^4^ N/m^2^ represents a severe reduction to merely 0.1% to 1% of the healthy stiffness, indicating a complete failure of the joint. To evaluate the performance of the proposed indicator, the joint stiffness *k* in the analytical model is varied across this logarithmic spectrum. The dynamic increments $\varepsilon^{dyn}\left(t\right)$ are extracted directly from the analytical solutions. Figure 4 illustrates the evolution of the PCC as a function of the joint stiffness.

As observed in Figure 4, a comparative analysis is conducted between the traditional total-strain correlation and the proposed dynamic-increment PCC across various health states. The proposed dynamic-increment indicator demonstrates a strictly monotonic and highly sensitive decline as the transverse stiffness deteriorates. Taking the baseline stiffness of a healthy joint $K_{0}$ as 5 × 10^6^ N/m^2^, the dynamic indicator descends from 0.96 at the intact state down to approximately 0.12 when the stiffness plummets to 1 × 10^5^ N/m^2^, a complete failure state. This extensive margin of variation and the monotonicity establish a one-to-one mapping between the indicator value and the joint stiffness. Particularly noteworthy is the steep gradient exhibited by the dynamic curve during the early-to-mid stages of structural degradation (from 5 × 10^6^ N/m^2^ down to 1 × 10^6^ N/m^2^), which proves that the proposed dynamic-increment PCC can sharply capture incipient joint damage.

By contrast, the traditional total-strain correlation curve exhibits plateau regions in both the initial high-stiffness regime and the terminal low-stiffness failure zone, meaning it fundamentally lacks a one-to-one mapping with the structural state. From the baseline health state down to complete failure, the traditional indicator merely decreases from 0.96 to 0.49, demonstrating a heavily compressed sensitive margin. Due to the severe static masking effect, the traditional curve remains virtually flat and numb during the early and middle degradation phases. A larger slope emerges only after the stiffness drops below 1 × 10^6^ N/m^2^, a severe damage state where the joint has already lost 80% of its initial healthy stiffness. This vividly demonstrates that the total-strain correlation is inherently blind to early-stage deterioration.

It should be acknowledged that the derived analytical dual-beam model operates under several idealized assumptions, such as omitting damping, boundary condition flexibility, and vehicle–bridge interactions (VBIs). While these physical complexities alter the exact waveforms and absolute amplitudes of the strain responses in real-world structures, they do not compromise or invalidate the core mechanical conclusion demonstrated by this baseline framework: the isolation of high-frequency dynamic increments inherently eliminates the dominant static masking effect. The structural implications of transverse load redistribution and damping will be scaled and verified through the numerical simulation experiments presented in Section 5.

### 4.3. Performance Under Varying Vehicle Velocities

**Figure 5:**
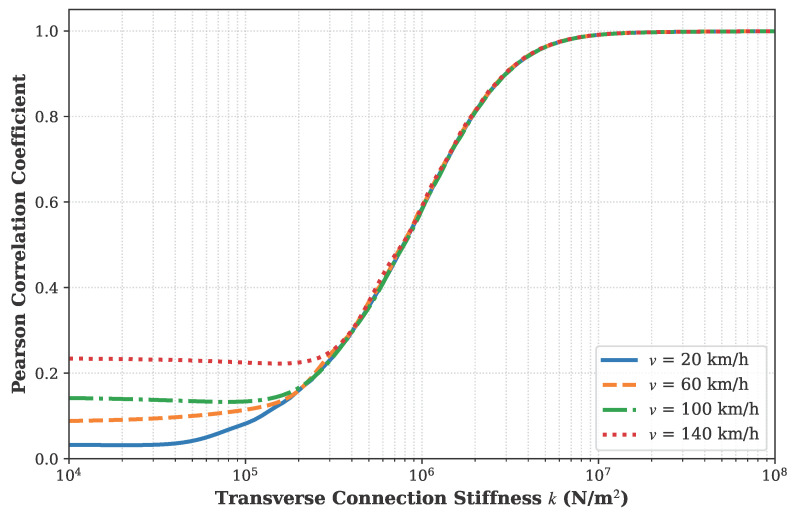
Evolution of the dynamic increment correlation with transverse connection stiffness under varying vehicle velocities.

Figure 5 illustrates the evolution of the dynamic-increment PCC under four representative vehicle velocities (spanning from 20 km/h to 140 km/h). As depicted, in the moderate damage, slight degradation and healthy stages (*k* > 1 × 10^6^ N/m^2^), the correlation curves under different velocities closely coincide. This consistency demonstrates that the dynamic-increment PCC is robust against traffic speed variations within these operational stages.

The influence of vehicle velocity only becomes discernible in the terminal regimes. The asymptotic minimum value of the PCC exhibits a slight velocity-dependent dispersion, where higher speeds result in marginally elevated residual correlations. However, since the fundamental objective of SHM is the timely detection of incipient faults, this late-stage dispersion does not compromise the metric’s practical utility. On the contrary, the immunity to velocity variations during the early degradation phases solidifies its reliability and superiority as an early-warning diagnostic tool.

### 4.4. Robustness to Vehicle Weight Variations

**Figure 6:**
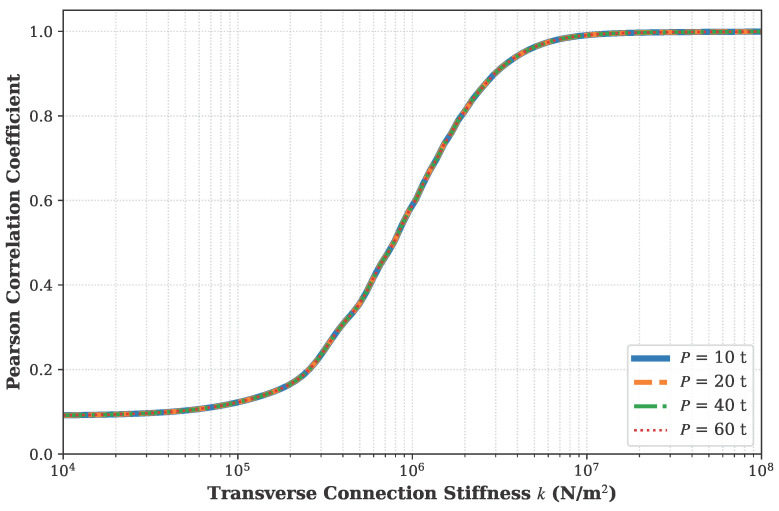
Evolution of the dynamic increment correlation with transverse connection stiffness under different vehicle load magnitudes.

To verify the robustness to load magnitude variations, the dynamic-increment PCC curves are calculated under four typical gross vehicle weights: 10 t, 20 t, 40 t, and 60 t. As depicted in Figure 6, the PCC curves under varying vehicle loads perfectly coincide. This overlap physically originates from the linearity of the system and the scale-invariance property of the PCC. While an increase in the vehicle weight *P* proportionally amplifies the dynamic strain responses, it does not alter the underlying modal synchronization and phase disparity governed by the hinge joint stiffness *k*. Consequently, the load-induced amplitude scaling is neutralized during the normalization process of the correlation calculation.

In summary, the parametric analyses conducted in this section reveal that the proposed dynamic increment correlation possesses three properties: sensitivity to hinge stiffness degradation, consistent performance across varying vehicle velocities, and theoretical invariance to vehicle load magnitudes. These characteristics collectively establish the primary advantage of the proposed method: it functions as an external-insensitive, structural-intrinsic indicator, which provides a reliable mapping of the transverse connection condition within the numerical context, and demonstrates numerical feasibility for the automated, long-term monitoring of PMGBs.

## 5. FE Simulation Verification

### 5.1. FE Modeling and Stochastic Traffic Simulation

In this study, the numerical simulations are built upon the PMGB model and the stochastic traffic load model developed in the previous research [14,15].

#### 5.1.1. FE Model of the Baseline PMGB

**Figure 7:**
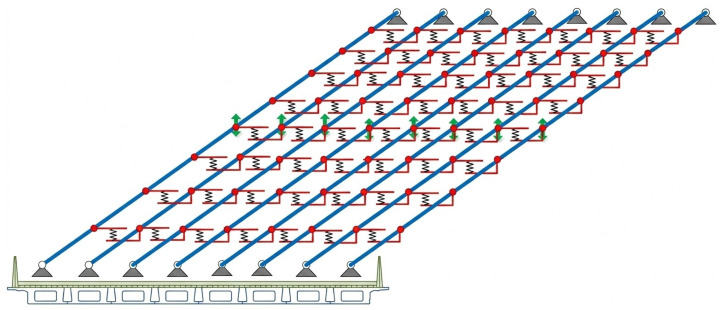
Mechanical modeling topology of the simply supported PMGB. In this schematic, the blue lines represent the longitudinal precast girders, the black springs denote the equivalent vertical shear springs for the hinge joints, the red dots and transverse lines indicate the finite element nodes and rigid arms, and the green arrows highlight the mid-span locations where structural responses are monitored. (Note: For visual clarity, the longitudinal mesh density depicted in the schematic has been coarsened; the actual numerical model utilizes 20 elements per slab.)

A generalized schematic illustrating the topological modeling approach is depicted in Figure 7. Specifically, the numerical simulations in this study are conducted on a baseline bridge model comprising 8 identical precast hollow slabs. The geometric dimensions and material properties of each hollow slab are detailed in Table 2.

To simulate the shear-transfer mechanism of the hinge joints, adjacent slabs are coupled using vertical shear springs. The baseline healthy stiffness of these continuous springs, denoted as $K_{0}$, is set to 5000 kN/m^2^. This value is rigorously determined based on the equivalent shear deformation compatibility of the joint concrete. It ensures that the lateral load distribution factors of the numerical model align with the macroscopic behavior of intact PMGBs, as validated by previous studies [16,22,23]. Furthermore, a Rayleigh damping matrix is employed to model the energy dissipation of the structure, where the damping ratios for the first and third bending modes are set to 3%.

Finally, the continuous structural system is spatially discretized for numerical computation. Each longitudinal slab and its corresponding hinge joints are divided into 20 equidistant elements connected by 21 nodes. Accordingly, the distributed linear stiffness $K_{0}$ is subsequently converted into discrete nodal spring stiffnesses based on the element length.

#### 5.1.2. Stochastic Traffic Load Model

The dynamic excitation is generated using a stochastic traffic load model, which is established under the following simplifications: (1) the traffic load is approximated as a stationary random process; (2) each vehicle is idealized as a moving concentrated force traveling uniformly across the bridge; and (3) each vehicle is parameterized by its generation probability, transverse position, traveling speed, and gross weight.

Specifically, the vehicle arrival process is modeled by a Poisson distribution with a rate parameter of λ=0.05, indicating an average vehicle entry interval of approximately 20 s. Within a given lane, the lateral positions of the vehicles are assumed to follow a normal distribution with a standard deviation of 0.3 m relative to the lane centerline. The traveling speeds are also normally distributed with a mean of 80 km/h and a standard deviation of 10 km/h.

To accurately reflect the complex composition of real-world traffic, the random vehicle weight, denoted as *w*, is formulated using a three-peak mixture distribution. The weights of small and medium vehicles are characterized by normal distributions, whereas the weights of heavy trucks (large vehicles) are described by an extreme minimum value distribution (e.g., Gumbel distribution). The overall probability density function f(w) is expressed as:

(26) $$f\left(w\right)=p_{1}\frac{1}{\sigma_{1}\sqrt{2\pi }}e^{-\frac{{\left(w-\mu_{1}\right)}^{2}}{2\sigma_{1}^{2}}}+p_{2}\frac{1}{\sigma_{2}\sqrt{2\pi }}e^{-\frac{{\left(w-\mu_{2}\right)}^{2}}{2\sigma_{2}^{2}}}+p_{3}\frac{1}{\beta }e^{-\frac{w-\alpha }{\beta }-e^{-\frac{w-\alpha }{\beta }}},$$

where $p_{1}=0.65$, $p_{2}=0.2$, and $p_{3}=0.15$ denote the traffic proportions of small, medium, and large vehicles, respectively. For small and medium vehicles, the mean weights are set to $\mu_{1}=2$ t and $\mu_{2}=15$ t, with corresponding standard deviations of $\sigma_{1}=0.5$ t and $\sigma_{2}=4$ t. For the large vehicles, the extreme value distribution is defined by a location parameter α=42 t and a scale parameter β=5 t.

### 5.2. Demonstration of the Streaming Processing Pipeline

To verify the effectiveness of the framework proposed in Section 3.3, a stochastic traffic flow continuing for 12 h is applied to the baseline PMGB model. The dynamic strain responses of the mid-span cross-section are extracted at a sampling rate of 50 Hz. The execution of the streaming pipeline is demonstrated through three sequential stages: single-vehicle crossing event extraction, high–low frequency strain separation, and indicator calculation.

#### 5.2.1. Single-Vehicle Crossing Extraction

**Figure 8:**
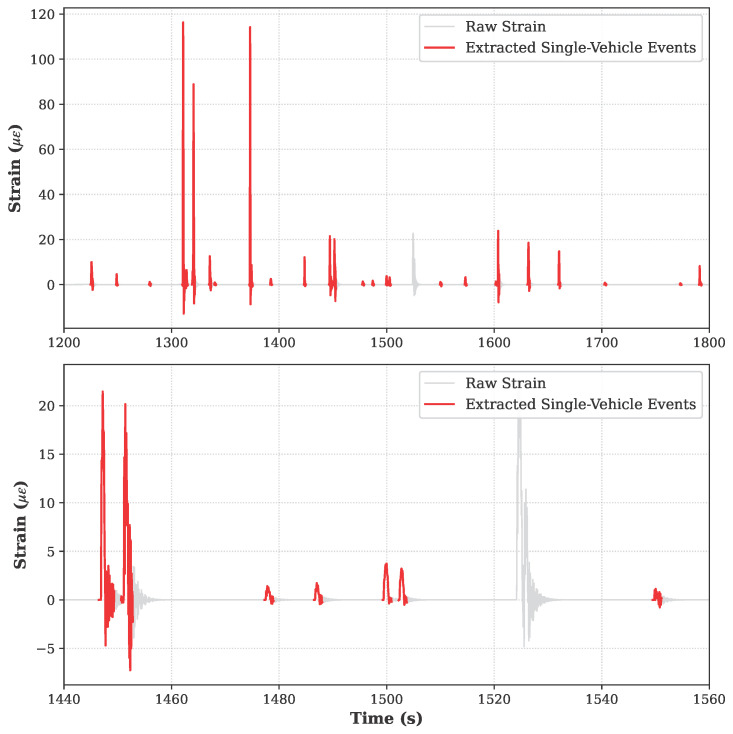
Demonstration of the event extraction for Girder 1: the highlighted region represents the complete single-vehicular event captured by the dynamic state machine, with a localized zoomed-in view confirming the successful exclusion of a closely-spaced multi-vehicle event.

Figure 8 illustrates a segment of the continuous dynamic strain response generated by the stochastic traffic, where the extracted single-vehicle crossing events are highlighted in red. As explicitly demonstrated in the zoomed-in view, a complex multi-vehicle event, where two vehicles enter the bridge almost simultaneously (around 1525 s), is successfully excluded by the algorithm. Meanwhile, other single-vehicle events are identified and retained. Unlike conventional fixed-window slicing methods that often suffer from data fragmentation, the proposed algorithm dynamically identifies the event boundaries. By incorporating the pre-trigger buffer and post-trigger cooldown mechanisms, the algorithm successfully captures the complete structural response without truncating the “head” or “tail” of the vehicle-induced excitation. In the simulation, 2165 vehicles are generated while 1963 single-vehicle events are extracted, indicating about 9.33% of the vehicles are discarded.

#### 5.2.2. High–Low Frequency Separation

**Figure 9:**
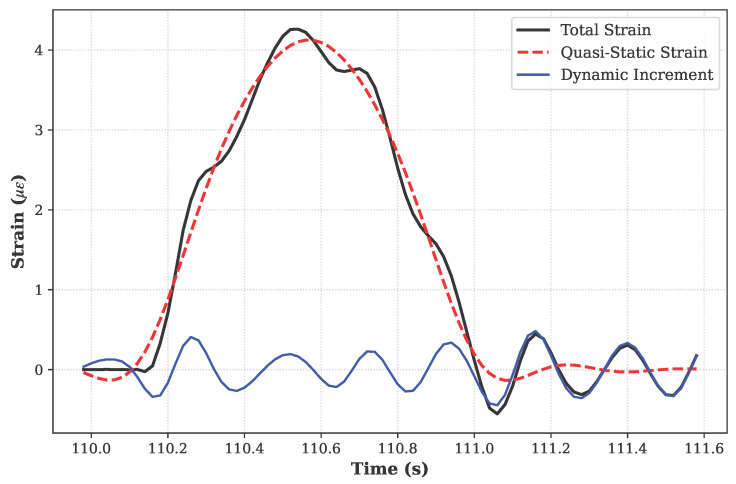
Decomposition of the raw strain response into the low-frequency quasi-static component and the high-frequency dynamic increment using the Ormsby filter.

Following the event interception, the Ormsby zero-phase filter [17] is utilized to decouple the quasi-static response from the high-frequency dynamic increment. As shown in Figure 9, taking the mid-span strain of Beam 6 during an event as an example, the raw extracted strain (black solid line) is decomposed. The massive quasi-static component (red dashed line) is primarily governed by the gross vehicle weight and the global structural redundancy, whereas the extracted high-frequency dynamic increment (blue solid line) intrinsically contains the localized modal interaction information across the transverse connections.

#### 5.2.3. Indicator Calculation

**Figure 10:**
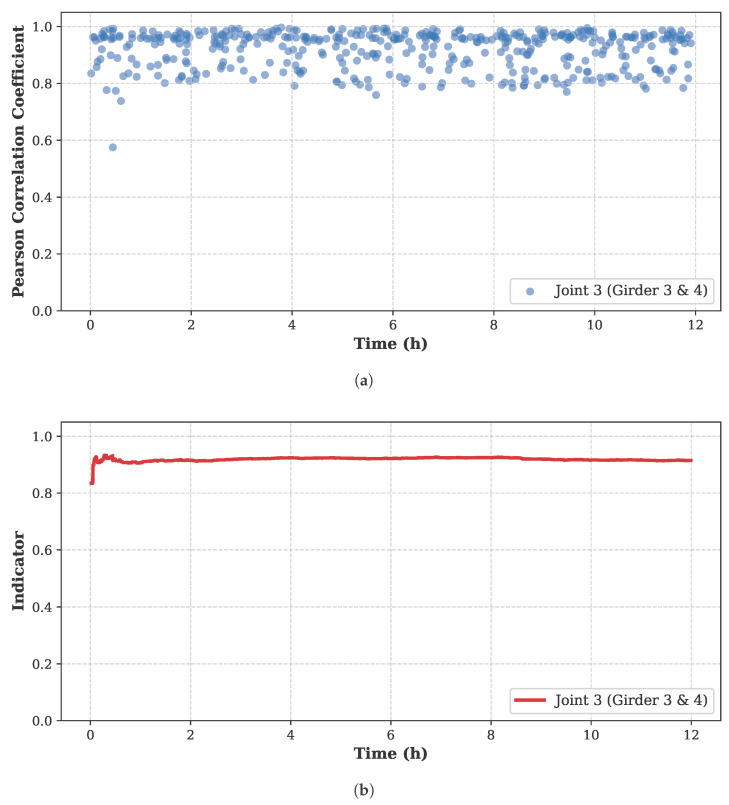
Demonstration of the indicator calculation: (**a**) raw instantaneous correlation coefficients exhibiting severe stochastic fluctuations; (**b**) the convergent and stable baseline indicator extracted via adaptive smoothing.

Finally, the PCCs of the dynamic strain increments between adjacent girders are computed. Owing to the highly stochastic nature of the operational traffic flow (variations in gross weights, driving speeds, and lateral lane positions), the instantaneous PCCs derived from isolated vehicle crossing events naturally exhibit severe statistical dispersion. As depicted in Figure 10a, these raw data points fluctuate across the temporal domain. Such variance is highly undesirable for SHM, as an ideal condition indicator must be intrinsically governed by the physical state of the structure rather than being overshadowed by the randomness of transient external excitations.

To mitigate this excitation-induced variance, a moving average smoothing mechanism is implemented, as formulated in Equation (25). This procedure effectively filters out the stochastic uncertainty inherent in individual vehicular events. As demonstrated in Figure 10b, the scattered data is successfully transformed into a continuously convergent and stable baseline indicator curve.

In this simulation, the indicator typically achieves a stable convergence within approximately one hour of data accumulation. Given the traffic flow parameters (an average arrival interval of 20 s), roughly 180 vehicle-crossing events occur per hour. Consequently, setting the moving average window length *N* to 200 is a conservative and reliable approach to ensuring the statistical stability of the baseline indicator. This converged baseline effectively decouples the structural behavior from the traffic uncertainties, thereby serving as a robust benchmark for tracking hinge joint degradation and identifying early-stage damage.

### 5.3. Scenario A: Global Synchronous Degradation

In standard operational environments, adjacent precast girders are subjected to similar temperature cycles, material shrinkage, and general long-term material aging. This naturally leads to a global synchronous degradation of all hinge joints across the bridge deck. To simulate this scenario, the stiffness of all hinge joints is symmetrically and uniformly reduced from the intact state (α=0.0) down to a complete failure state (α=1.0). Identical stochastic traffic flow is utilized across all reduction stages to rigorously eliminate any traffic-induced statistical discrepancies.

**Figure 11:**
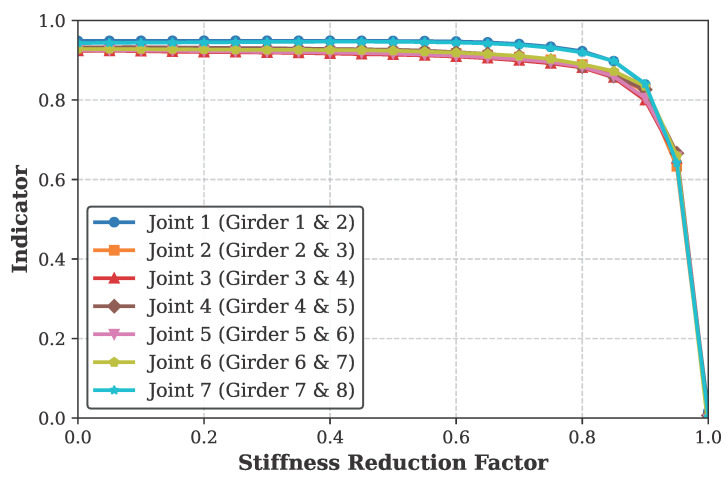
The evolutionary trajectories of the proposed indicators across all seven hinge joints under global synchronous degradation.

Figure 11 illustrates the evolutionary trajectories of the proposed indicators for all seven hinge joints under global degradation. It can be observed that as the global stiffness reduction factor α increases from 0.0 to 1.0, the indicators for all joints exhibit a highly consistent and monotonic downward trend. Although minor spatial variations exist in their baseline magnitudes due to the varying lateral distances from the random traffic loads, the degradation patterns remain remarkably uniform. This demonstrates that the proposed indicator possesses the fundamental capability to characterize structural deterioration.

**Figure 12:**
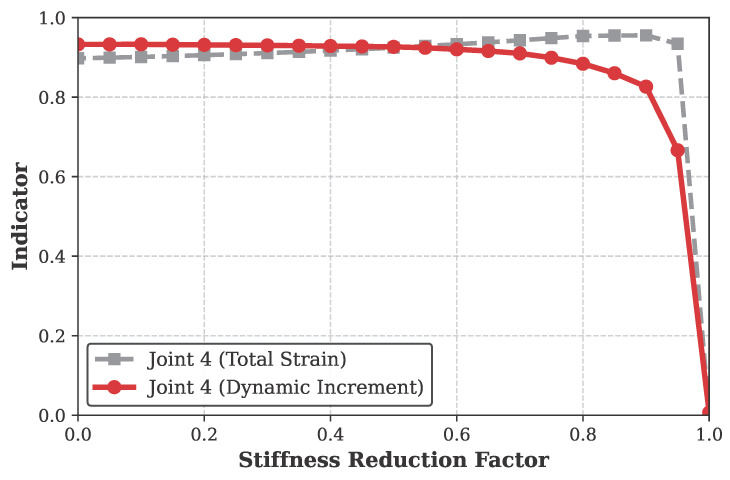
Sensitivity comparison for Joint 4 between the total-strain indicator and the proposed dynamic-increment indicator under the global synchronous degradation scenario.

Building upon this, Figure 12 presents a comparative analysis between the proposed dynamic-increment indicator and the conventional total-strain indicator for the central joint (Joint 4). Unlike the proposed indicator, the conventional indicator remains heavily insensitive to the global stiffness deterioration. This vivid contrast explicitly exposes the static masking effect, where the massive quasi-static deflections dominated by gross vehicle weights severely overshadow the asynchronous dynamic vibration.

Notably, a counter-intuitive phenomenon can be observed in the total-strain indicator’s trajectory: during the early-to-mid stages of stiffness degradation (as α increases from roughly 0.1 to 1.0), the indicator increases rather than decreases. This is fundamentally caused by the mechanical low-pass filtering effect of the degrading hinge joints. In a healthy state, the rigid joints effectively transmit out-of-phase high-frequency modal vibrations, which slightly reduces the overall waveform similarity. However, as the joints soften, the transverse transmission of high-frequency dynamic components is significantly attenuated, leaving the responses of adjacent girders dominated almost exclusively by the in-phase quasi-static components. Consequently, the PCC of the total strain falsely rises, explicitly demonstrating the unreliability of utilizing total-strain correlation for damage detection.

### 5.4. Scenario B: Localized Joint Degradation

In real-world expressway operations, the right-side slow lanes are predominantly subjected to massive cyclic loads from heavy freight trucks. Consequently, the hinge joints connecting the precast slabs directly beneath these lanes are significantly more vulnerable than other joints. For a standard bridge deck layout accommodating two driving lanes and an emergency shoulder, Joints 4 and 5 are typically the most susceptible to such localized damage. To replicate this practical condition, Scenario B simulates a concurrent progressive degradation on both Joints 4 and 5, while the stiffness of all remaining joints is kept intact.

**Figure 13:**
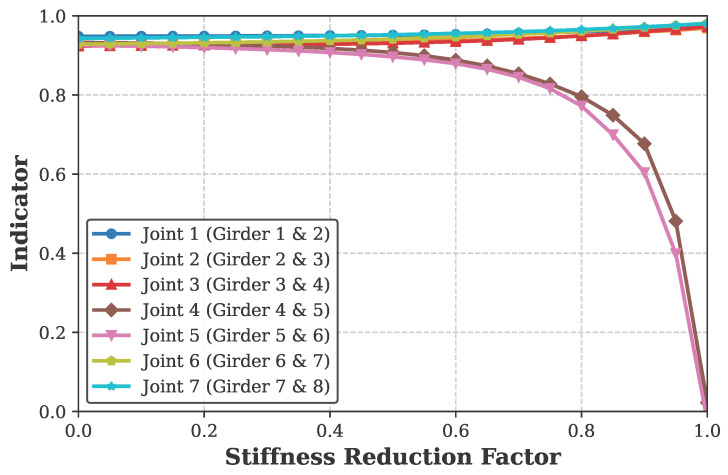
The evolutionary trajectories of the proposed indicators under localized multi-joint degradation. The algorithm exhibits spatial isolation, triggering sensitive responses exclusively for the damaged joints (Joints 4 and 5) while maintaining stability for the healthy joints.

Figure 13 reveals the evolutionary trajectories of the proposed indicators under this localized degradation scenario. As the local stiffness reduction factor α of the damaged joints increases from 0.0 to 1.0, the indicators for Joint 4 and Joint 5 exhibit monotonic declines. Remarkably, the indicators for all healthy joints (i.e., Joints 1–3, 6, and 7) securely remain stable at their baseline healthy levels. This performance validates that the proposed algorithm possesses excellent spatial isolation capabilities, ensuring that localized deterioration does not mechanically contaminate the condition assessment of the surrounding healthy connections, thereby effectively preventing false alarms.

**Figure 14:**
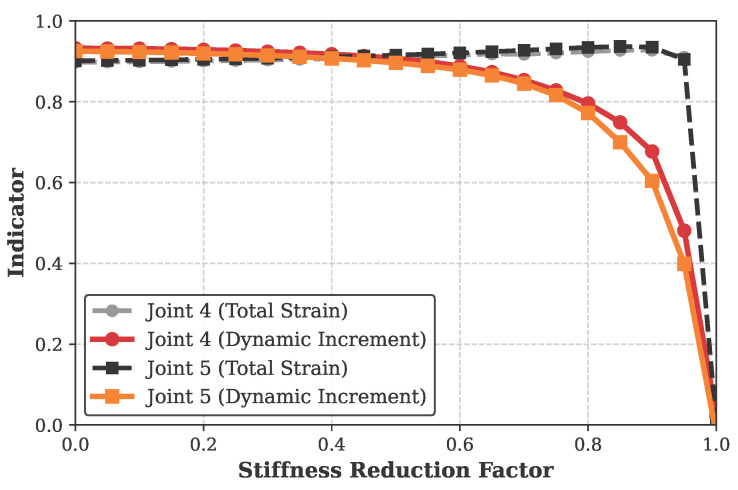
Sensitivity comparison for the damaged joints (Joints 4 and 5) between the total-strain and dynamic-increment indicators.

Furthermore, Figure 14 presents a comparative analysis between the dynamic-increment and total-strain indicators for the damaged joints. Similarly to the global synchronous degradation scenario, the total-strain indicators fail to capture the localized degradation until the joints are nearly entirely ruptured. By decoupling the high-frequency components, the proposed indicator provides effective identification of localized damage.

### 5.5. Scenario C: Sudden Severe Localized Damage

In addition to progressive fatigue aging, hinge joints are highly vulnerable to sudden severe damage triggered by extreme events. A typical and highly dangerous scenario occurs when extremely overweight trucks occupy the edge lane. In engineering practice, such catastrophic chain reactions, where hinge joint deterioration degenerates into “single-slab behavior” followed by sudden collapse under extreme overloading, have been witnessed in real-world accidents, including the 2005 Lake View Drive Bridge collapse in the United States [3] and the 2011 Qianjiang Third Bridge collapse in China [13].

Generally, an edge lane comprises two to three precast girders. Under massive eccentric overloading, these edge girders tend to deflect downwards as a cohesive block. Consequently, an immense forced differential shear is instantaneously concentrated at the hinge joint between the overloaded edge lane and the adjacent mid lane, resulting in sudden severe damage. To simulate this state, a simulation is conducted over a 12.0 h operation period. Exactly at *t* = 4.0 h, sudden damage occurs in Joint 2, causing its stiffness to plummet to 20% of $K_{0}$ (α=0.8). This represents a critical state where severe concrete cracking and reinforcement yielding have occurred, placing the joint on the verge of structural rupture, yet still retaining a marginal friction-based load transfer capacity before an imminent collapse.

**Figure 15:**
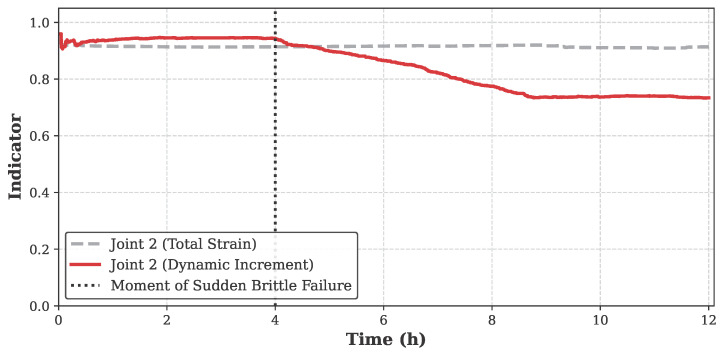
Real-time monitoring timeline of a sudden severe failure at Joint 2 (α jumps abruptly to 0.8).

As illustrated in Figure 15, immediately following the sudden structural damage at the 4th hour, the slope of the proposed indicator experiences an obvious descent. This slope discontinuity serves as a numerical signature for potential pre-collapse early warning frameworks. Conversely, the total-strain indicator fails to register this critical event. As demonstrated in previous scenarios, at α=0.8, the total-strain indicator is still obscured by the static masking effect, remaining in the high-correlation zone (near 0.9). This contrast demonstrates that the introduced high-low frequency decoupling is a critical necessity for achieving sensitive anomaly detection before actual structural failure.

**Figure 16:**
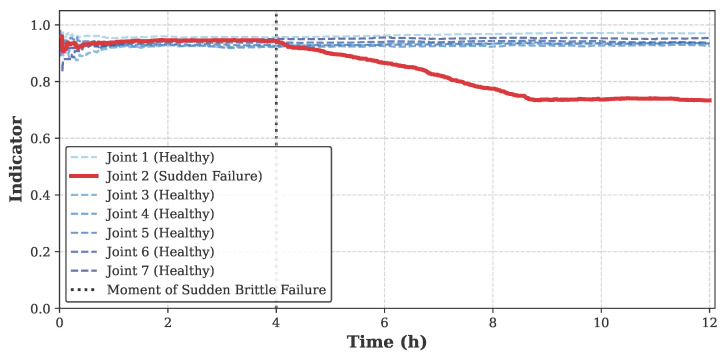
Temporal evolution of the proposed indicators for all hinge joints under the sudden severe damage scenario.

Furthermore, to verify the damage localization capability of the proposed algorithm, the trajectories of all hinge joints are plotted in Figure 16. It is observed that while the indicator for the critically damaged Joint 2 drops substantially, the indicators for all remaining healthy joints maintain their baseline values, unaffected by the localized failure.

In summary, by monitoring the discontinuity in the indicator’s slope, the proposed framework establishes a theoretical and numerical foundation for detecting and localizing sudden severe damage, supporting the future development of online emergency warning systems.

### 5.6. Robustness Against Measurement Noise

In operational bridge monitoring, strain measurements are inevitably contaminated by noise. A critical inherent vulnerability of the PCC is its sensitivity to uncorrelated broadband noise. Mathematically, the injection of independent white noise inflates the variance (the denominator of the PCC formula) without contributing to the covariance, thereby suppressing the baseline value of the indicator. Therefore, evaluating the noise boundary of the proposed algorithm is crucial for its practical deployment.

**Figure 17:**
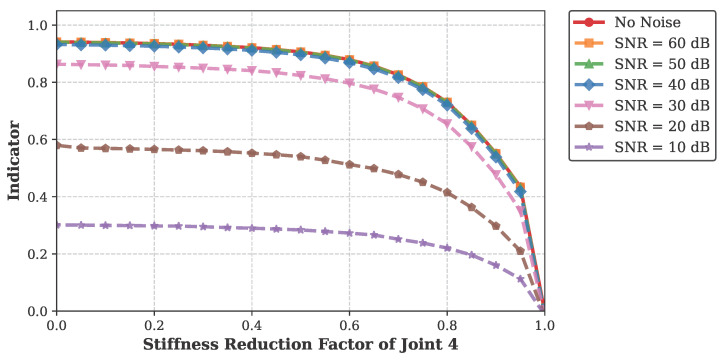
Noise robustness evaluation of the proposed indicator under varying SNRs. The algorithm maintains robust representation capability at 30 dB, while defining 20 dB as the critical failure boundary for the dynamic feature extraction.

To investigate this limitation, the localized degradation scenario on Joint 4 is re-evaluated under a range of Signal-to-Noise Ratios (SNRs): Clean, 60 dB, 50 dB, 40 dB, 30 dB, 20 dB, and 10 dB. As illustrated in Figure 17, when the SNR is greater than or equal to 40 dB, the noise has virtually no impact on the proposed indicator, with the evolutionary trajectories overlapping the clean baseline. When the SNR decreases to 30 dB, the indicator experiences a slight baseline reduction but remains functional and acceptable for damage detection. However, when the SNR drops to 20 dB and below, the indicator trajectory becomes severely distorted. Despite the integrated filtering process, the uncorrelated in-band noise energy becomes comparable to the dynamic increments, causing the healthy baseline to plummet and compressing the recognizable margin for damage characterization.

### 5.7. Robustness Against Hyper-Parametric Selection

To establish the practical engineering utility of the proposed framework, a quantitative sensitivity analysis is conducted in this section to evaluate the algorithmic robustness against variations in key hyper-parameters, including the vehicle crossing event extraction hyper-parameters, the filter cut-off frequencies, and the indicator smoothing length.

#### 5.7.1. Vehicle Crossing Event Extraction Hyper-Parameters

**Figure 18:**
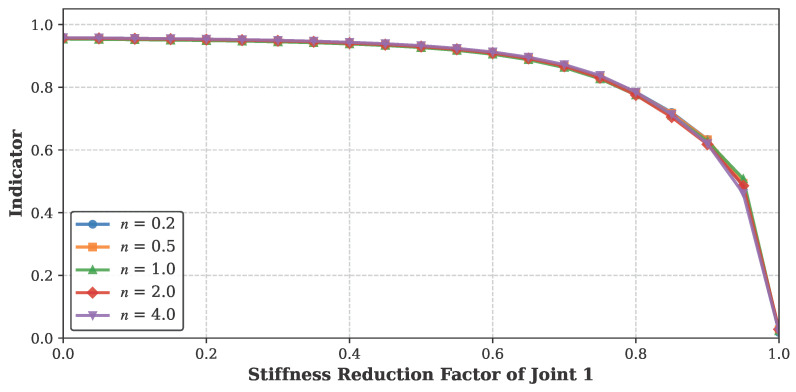
Evaluation of the converged indicator under varying MWA window length multiples (*n*) across the entire stiffness degradation lifecycle.

The automated vehicle crossing event extraction procedure is governed by two key hyper-parameters: the moving-average window length and the event-triggering amplitude threshold. The MWA window length *W* is scaled to match a multiple of the bridge’s fundamental period, defined as $W=\left\lfloor \frac{n}{f_{1}}\;\cdot \;F_{s}\right\rfloor$, where $f_{1}$ represents the fundamental frequency of the bridge, and $F_{s}$ denotes the sampling frequency. Theoretically, selecting an integer period multiple (such as the baseline configuration of n=2.0) forces the average of the dynamic increments within the window to approach zero. This allows the global MWA envelope to approximate the quasi-static component, thereby enabling an accurate rising- and falling-edge trigger. To evaluate the algorithmic robustness against sub-optimal parameter tunings, the window multiple was varied across a broad range n∈{0.2,0.5,1.0,2.0,4.0}. As demonstrated in Figure 18, the converged indicator values across all five multipliers are remarkably superimposed throughout the entire joint degradation lifecycle. This explicitly demonstrates that the final converged indicator is highly independent of the window length.

The triggering threshold γ acts as the secondary detector within the processing pipeline to isolate vehicle-induced structural responses from ambient states. Theoretically, variations in this threshold introduce two influences on the extraction framework.

**Figure 19:**
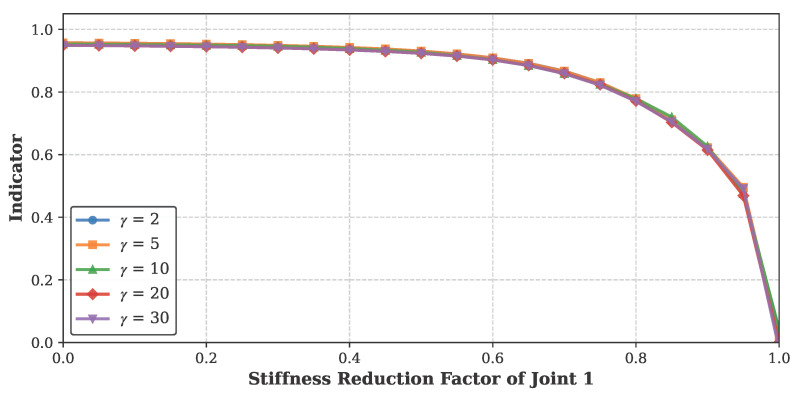
Evaluation of the indicator under varying triggering thresholds across the entire stiffness degradation lifecycle.

First, for an isolated single-vehicle crossing event, modifying the threshold directly alters the truncated temporal length of the extracted signal (i.e., the data points captured at the head and tail of the event), which could potentially perturb the calculated correlation. However, as illustrated in Figure 19, the proposed indicator exhibits excellent robustness against this truncation variation. The evolution trajectories of the indicator under different thresholds (γ∈{2,5,10,20,30}ε) are nearly superimposed across the entire structural degradation lifecycle.

**Figure 20:**
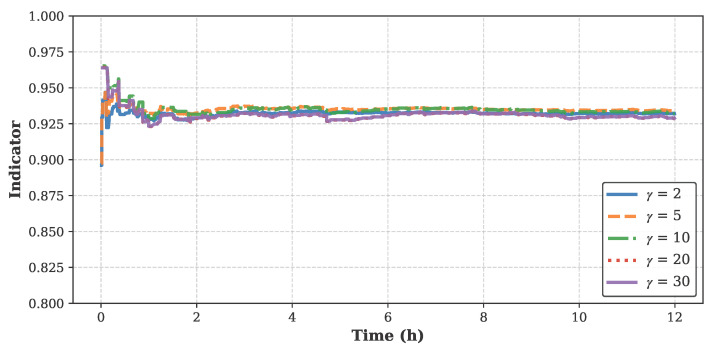
Convergence processes of the indicator under varying triggering thresholds at a slight degradation state (3 × 10^6^ N/m^2^).

Second, setting a higher threshold might completely filter out the weak structural responses induced by lightweight cars. This mechanism restricts the data pool exclusively to heavier vehicles, reducing the total number of captured crossing events. Because fewer events are fed into the MWA calculation, the transient convergence process of the indicator could theoretically be affected. Nevertheless, as presented in Figure 20, this impact is almost negligible over continuous monitoring. Although slight localized statistical divergences occur during the initial phase (0 to 2 h) due to differing event capture rates, all indicator trajectories robustly converge to an identical steady-state baseline.

In summary, the proposed method demonstrates exceptional robustness toward the event extraction hyper-parameters (*n* and γ). This performance is rooted in the intrinsic properties of the processing pipeline: the mathematical scale invariance of the PCC neutralizes amplitude discrepancies stemming from different vehicle weights or edge truncation depths, ensuring that the indicator remains structurally intrinsic. Finally, it is worth noting that while the framework handles loose parameter tolerances, γ must not be configured excessively low to prevent false triggers from random environmental noise floors.

#### 5.7.2. Filter Cut-Off Frequencies

**Figure 21:**
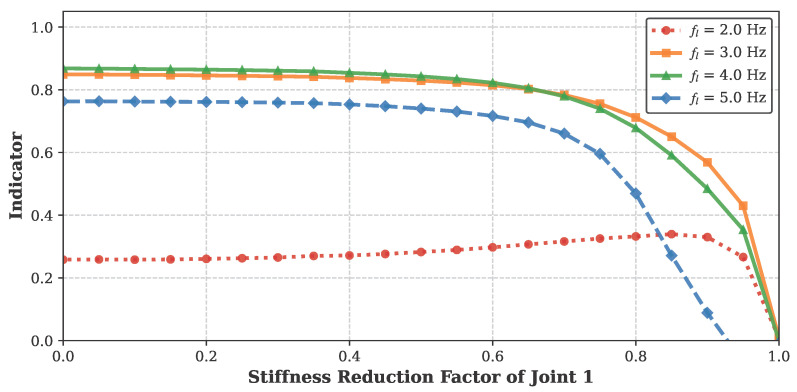
Evaluation of the indicator under varying filter cut-off frequencies ($f_{l}$) across the entire stiffness degradation lifecycle.

The execution of the signal separation pipeline relies on the frequency-targeted Ormsby filter to decouple damage-sensitive dynamic increments from the energy-dominant quasi-static responses. In this simulation with a mean speed of 80 km/h (σ=10 km/h), the 3σ upper bound velocity reaches 110 km/h (30.56 m/s). Given the girder length of 20 m, the 1st-order quasi-static driving frequency is approximately 0.76 Hz. To cleanly strip away the quasi-static components, the higher-order quasi-static harmonics up to the 3rd harmonic (about 2.28 Hz) should be removed, while the fundamental modal frequency of the bridge ($f_{1}\approx 4.563$ Hz) must be preserved. To identify the optimal cut-off frequency, a scan across a frequency range of 2.0 Hz to 5.0 Hz was conducted, as illustrated in Figure 21.

When a low cut-off frequency of $f_{l}=2.0$ Hz is adopted, the filter fails to isolate the higher-order quasi-static components (specifically the third-order and above). Due to the spatial low-pass filtering characteristic of the hinge joint as demonstrated in Section 2.3, these higher-frequency quasi-static responses cannot be effectively transmitted from the loaded girder to the adjacent unloaded girders. This transmission block induces waveform misalignment within the filtered signals. Consequently, the PCC of these leaked quasi-static components remains extremely poor, forcing the indicator to drop to an abnormally low baseline.

When $f_{l}$ is configured at 3.0 Hz or 4.0 Hz, the filter successfully attenuates the primary energy of the quasi-static strain while fully preserving the dynamic strain increments. The curves corresponding to these two cut-off frequencies are highly coincident, maintaining a robust, monotonic, and uniform descending sensitivity with respect to the joint stiffness reduction.

Finally, when $f_{l}$ is set to 5.0 Hz, thereby surpassing the structural fundamental frequency ($f_{1}\approx 4.56$ Hz), the 1st-order modal components that possess the dominant vibration energy are truncated. Although the remaining dynamic increments extracted from higher-order modes still reflect the trend of stiffness reduction, the absolute response amplitudes of these high-order modes are substantially small. As a result, the signals become highly susceptible to ambient noise interference, causing the indicator curves to shift downward relative to the optimal baselines ($f_{l}=3.0$ Hz or 4.0 Hz).

In summary, the operational configuration of the filter cut-off frequency should be set sufficiently high to remove higher-order quasi-static components, yet remain below the fundamental eigenfrequency of the bridge to incorporate the major modal energy, thereby maintaining both indicator effectiveness and noise robustness.

#### 5.7.3. Indicator Smoothing Length

The moving smoothing window length *N* in Equation (25) is a critical hyper-parameter that governs the trade-off between the statistical stability of the indicator and the real-time performance in response to sudden damage. The primary objective of implementing *N* is to suppress the statistical fluctuations induced by stochastic traffic, thereby transforming the highly random single-event PCCs into a steady, structurally intrinsic indicator.

**Figure 22:**
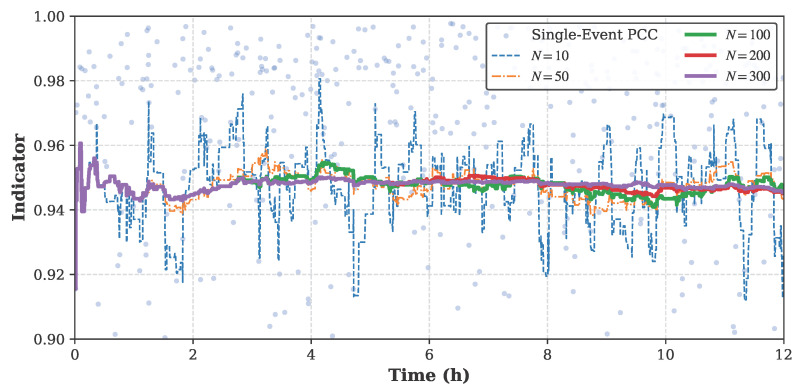
Continuous convergence processes and background raw single-event PCC distributions under varying smoothing window lengths (*N*).

Figure 22 illustrates the curves of the indicator across different smoothing lengths (N∈{10,50,100,200,300}) superimposed on the raw single-event PCCs. As observed, insufficient smoothing lengths (N≤100) are inadequate to achieve statistical convergence; the resulting curves fluctuate heavily in response to the random vehicle loads, which risks misjudgment of the true structural condition. In contrast, when *N* is increased to 200 or 300, the indicators attain steady-state convergence, confining the fluctuations within a narrow band. This subtle variation is negligible and does not impair the structural condition assessment, proving that the indicator has decoupled from loading stochasticity and stabilized into an inherent structural property.

**Figure 23:**
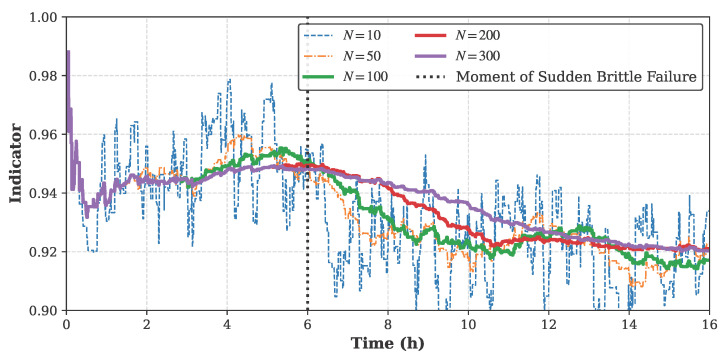
Transient tracking trajectories under a sudden stiffness step-drop at t=6 h under varying smoothing window lengths.

However, an oversized window penalizes the indicator’s sensitivity to sudden joint damage. Figure 23 presents the transient tracking performance under an instantaneous 50% joint stiffness step-drop introduced at t=6 h. As observed, among the curves (N∈{100,200,300}), a smaller window length *N* exhibits a steeper descending slope following the onset of damage. The excessive historical memory embedded within N=300 compromises the tracking sensitivity, causing a sluggish transition zone and introducing undesirable warning latencies.

In summary, the smoothing length *N* should be selected as small as possible, provided that statistical convergence is rigorously satisfied. Consequently, N=200 is established as a well-balanced configuration for the proposed framework. It successfully maximizes the tracking sensitivity and slope sharpness for sudden joint damage while safeguarding the statistic stability of the proposed indicator.

## 6. Conclusions

This study proposes a novel indicator for the monitoring and evaluation of transverse connection condition in PMGBs based on the correlation of high-frequency dynamic strain increments. By addressing the inherent limitations of conventional total-strain indicators through theoretical derivation and numerical verification, the performance of the proposed framework is evaluated.

### 6.1. Main Conclusions

The primary findings of this study are summarized as follows:

1. **Elimination of the Static Masking Effect:** Theoretical analysis reveals that the insensitivity of the total-strain indicator stems from the static masking effect, where high-energy, in-phase quasi-static components overshadow the subtle, asynchronous dynamic oscillations triggered by damage. By utilizing the Ormsby band-pass filtering strategy, the proposed method effectively strips away the quasi-static baseline, restoring the indicator’s sensitivity to hinge joint deterioration.
2. **Robustness to Traffic Randomness:** Parametric studies demonstrate that the proposed dynamic-increment indicator is an external-insensitive, structural-intrinsic metric. It exhibits theoretical invariance to variations in vehicle weight due to the scale invariance of the PCC and maintains consistent performance across operational speed ranges (20 to 140 km/h), ensuring reliable long-term monitoring under stochastic traffic flows.
3. **Dual-mode Monitoring and Spatial Localization:** The framework effectively addresses dual engineering requirements: providing quantitative support for long-term proactive maintenance (Scenarios A and B) and providing a numerical basis for future emergency warning systems against sudden brittle failures (Scenario C). Furthermore, the algorithm demonstrates robust spatial isolation, accurately localizing damage to specific joints without triggering false alarms in adjacent healthy sections.
4. **Application Boundaries in Noisy Environments:** The proposed indicator is sensitive to measurement noise, particularly when the SNR drops below 30 dB. Consequently, 30 dB is identified as the critical operational boundary, highlighting the necessity of high-quality data acquisition and band-limited filtering for reliable field implementation.

### 6.2. Limitations

Despite the demonstrated robustness of the proposed framework, certain limitations of this study should be explicitly acknowledged. Most importantly, the current methodology relies purely on numerical validation and lacks validation using real-world bridge data from operational environments. This constraint stems from two primary practical challenges. First, capturing continuous sensor measurements that span the complete structural progression from incipient micro-cracking to severe collapse is practically prohibitive in the field due to safety risks. Second, physical laboratory experiments present technical hurdles in replicating realistic, long-term stochastic traffic flows.

Furthermore, the current FE model operates under idealized operational and sensing assumptions, omitting practical complexities that directly influence high-frequency strain increments. Specifically, VBI, pavement surface roughness, and multi-axle vehicular configurations inject wideband transient mechanical forces into the structure; these external high-frequency excitations can superimpose on the structural modal increments and amplify the statistical variance of single-event correlations. Regarding environmental and hardware constraints, while ultra-low-frequency temperature drift and sensor drift are effectively rejected by the Ormsby filter, severe sensor anomalies could introduce localized high-frequency artifacts. Most critically, multi-sensor synchronization errors represent a severe algorithmic vulnerability; because the dynamic-increment PCC fundamentally relies on instantaneous phase alignment, even minor timing asynchronies between adjacent sensors will artificially degrade the indicator and trigger false-positive damage evaluations.

A final limitation lies in the absence of a threshold system for translating the proposed indicator into actionable maintenance decisions (e.g., specific numerical thresholds for bridge closure or hinge retrofitting). Establishing reliable warning thresholds necessitates accumulating extensive empirical data over multi-year lifecycles, balanced against local socio-economic budgets and structural design codes.

### 6.3. Future Work

To bridge the gap between this theoretical baseline and complex field environments, our future work will proceed along two directions. First, the simulation models will be advanced to encompass pavement surface roughness, VBI, and realistic vehicular configurations, including multi-axle groups and dual-wheel transverse footprints.

Concurrently, next-phase research will apply the proposed streaming edge pipeline to long-term field monitoring data. By cross-referencing these continuous indicator curves with routine manual inspection results and emergency response reports, we aim to establish a multi-tier threshold system and evaluate its statistical reliability (e.g., false alarm rates). This will translate the proposed indicator into quantitative transverse connection conditions, supporting actionable maintenance decision-making.

In conclusion, the proposed high-frequency dynamic increment correlation framework shows numerical feasibility and offers a computationally efficient basis for the automated condition evaluation of PMGBs, supporting future field validation.

## Figures and Tables

**Table 1:** Parameters of the theoretical dual-beam model and moving vehicle.

Parameter	Value
Bridge Length	25 m
Material Density	2500 kg/m^3^
Cross-Section Area	0.9220 m^2^
Elasticity Modulus	32.5 GPa
Inertia Moment	0.4361 m^4^
Joint Stiffness	2000 kN/m^2^
Neutral Axis Distance	1.25 m
Vehicle Mass	10 t
Vehicle Velocity	20 m/s

**Table 2:** Default parameters of each precast hollow slab.

Parameter	Value
Bridge Length	20 m
Material Density	2500 kg/m^3^
Cross-Section Area	0.51 m^2^
Elasticity Modulus	34.5 GPa
Inertia Moment	0.05 m^4^
Hinge Joint Stiffness	5000 kN/m^2^
Neutral Axis Distance	0.45 m

## Data Availability

The source code presented in the study is openly available in GitHub at https://github.com/zhengwenhao03/Dynamic-Increment-Correlation (accessed on 22 June 2026).
